# Temporal aspects of copper homeostasis and its crosstalk with hormones

**DOI:** 10.3389/fpls.2015.00255

**Published:** 2015-04-17

**Authors:** Lola Peñarrubia, Paco Romero, Angela Carrió-Seguí, Amparo Andrés-Bordería, Joaquín Moreno, Amparo Sanz

**Affiliations:** ^1^Laboratory of Plant Molecular Biology, Department of Biochemistry and Molecular Biology, University of Valencia, ValenciaSpain; ^2^Department of Plant Biology, University of Valencia, ValenciaSpain

**Keywords:** *Arabidopsis thaliana*, *Oryza sativa*, copper homeostasis, copper transporters, hormone biosynthesis, hormone signaling, circadian clock, oxidative stress

## Abstract

To cope with the dual nature of copper as being essential and toxic for cells, plants temporarily adapt the expression of copper homeostasis components to assure its delivery to cuproproteins while avoiding the interference of potential oxidative damage derived from both copper uptake and photosynthetic reactions during light hours. The circadian clock participates in the temporal organization of coordination of plant nutrition adapting metabolic responses to the daily oscillations. This timely control improves plant fitness and reproduction and holds biotechnological potential to drive increased crop yields. Hormonal pathways, including those of abscisic acid, gibberellins, ethylene, auxins, and jasmonates are also under direct clock and light control, both in mono and dicotyledons. In this review, we focus on copper transport in *Arabidopsis thaliana* and *Oryza sativa* and the presumable role of hormones in metal homeostasis matching nutrient availability to growth requirements and preventing metal toxicity. The presence of putative hormone-dependent regulatory elements in the promoters of copper transporters genes suggests hormonal regulation to match special copper requirements during plant development. Spatial and temporal processes that can be affected by hormones include the regulation of copper uptake into roots, intracellular trafficking and compartmentalization, and long-distance transport to developing vegetative and reproductive tissues. In turn, hormone biosynthesis and signaling are also influenced by copper availability, which suggests reciprocal regulation subjected to temporal control by the central oscillator of the circadian clock. This transcriptional regulatory network, coordinates environmental and hormonal signaling with developmental pathways to allow enhanced micronutrient acquisition efficiency.

## Introduction

Mineral nutrition is an important environmental constraint that influences diverse developmental processes in plants. Both deficient and excess nutrient availability are considered abiotic stresses that can cause deleterious effects on plant physiology and metabolism. Plants employ complex homeostatic networks to increase uptake and cope with non-optimal nutrient supply. These mechanisms are especially relevant for transition metal nutrients, such as copper (Cu), since this metal acts as a double-edged sword in living beings. Cu is essential as a redox-active cofactor in multiple biological processes, but is toxic in excess given its role in the production of highly reactive oxygen species (ROS; Puig and Peñarrubia, 2009; Ravet and Pilon, 2013). Both local root responses and systemic signaling have to be integrated in order to drive optimized metal nutrient acquisition under changing environmental conditions which, in many cases, alter the whole plant morphology and metabolism (López-Bucio et al., 2003). In order to orchestrate morphological, physiological, and molecular adaptive responses to soil mineral bioavailabilities, phytohormones act as major endogenous cues. Nutrients affect multiple levels and components in hormone biosynthesis, perception, and signaling pathways. In turn, hormones influence root growth, stomatal movements and stress tolerance, among other processes, and have a huge impact on plant mineral nutrition. Thus, there is a close interrelation between hormonal stimuli and nutritional homeostasis (Rubio et al., 2009) which underlies current strategies based on co-application of hormones and mineral fertilizers to increase crop yield (Zaman et al., 2014).

Widespread inappropriate agricultural practices, such as overusing fungicides with high Cu concentrations, release of industrial wastewater and mining activities, have caused Cu contamination in cultivated soils and irrigating waters at specific locations (Marschner and Marschner, 2012). On the opposite side, the boost of carbohydrate synthesis in plants, due to the growing levels of atmospheric CO_2_, results in a general loss of the mineral nutritional quality of vegetable food. Indeed recent meta-analyses have indicated that Cu and other metal deficiencies would be exacerbated in forthcoming years due to a rise in CO_2_, with increasing obesity and “hidden hunger” problems in the world (Loladze, 2014; Myers et al., 2014). Since plants constitute one of the main entrances of micronutrients into trophic chains, deciphering the regulatory mechanisms underlying dynamical hormonal interactions with Cu uptake and distribution to edible plant parts is relevant for optimal plant development and to avoid plant nutritional deficiencies or excesses from being transferred to consumers.

Environmental factors, mainly light and temperature, show daily and seasonal variations, which impose a specific temporal order to the plant’s biological functions orchestrated by the circadian clock. Thus, hormone biosynthesis, perception and signaling pathways are under the control of the circadian clock, which explains its pervasive effects on plant growth and development (Robertson et al., 2009). Many abiotic stresses, such as cold, salt, drought and heat, result from daily light/dark cycles and the circadian clock influences responses to such stresses (Sanchez et al., 2011). Abiotic stress signaling pathways are highly interconnected because of common concurrent processes, where stress-related hormones are major components (Fujita et al., 2006). Plant nutrient requirements vary along daily cycles with plant development stages and during stress responses (Marschner and Marschner, 2012). The circadian regulation of ion channels and nutrient transporters involved in the transport of carbohydrates, nitrogen, sulfate, phosphate, and micronutrients is a pervasive phenomenon. These processes have been proposed to regulate downstream targets to further spread circadian signaling while, in turn, these processes provide feedback to the central oscillator (Ko et al., 2009; Haydon et al., 2011). Hormones can also affect time-of-day-dependent changes in metal fluxes, a phenomenon known as metal muﬄing. This term refers to the non-steady state dynamics of metal ions that involves temporal expression changes in homeostatic components, affecting uptake, eﬄux, and intracellular compartmentalization (Colvin et al., 2010). Due to its role in the basic metabolic processes determining the energy status of the plant (photosynthesis and respiration) Cu deficiency results in decreased plant growth. But its effects on reproductive growth are even more important. In cereals it leads to reduced pollen viability and increase in spikelet sterility, thus developing many unfilled grains and yield losses (Azouaou and Souvré, 1993; Dobermann and Fairhurst, 2000; Marschner and Marschner, 2012). But as increasing Cu concentrations may easily result in toxic effects, understanding the mechanisms that may optimize Cu use by the plant is a need. The study of those involved in achieving a coherent temporal integration of nutrient homeostasis and hormone responses will become increasingly relevant for food production. This is particularly important under the predicted effects of climate change on agriculture. Indeed it is around temperate agricultural where environmental stress conditions areas could have a massive impact on food production.

We herein collect disperse data on the interaction between Cu homeostasis and plant hormones, mainly abscisic acid (ABA) and ethylene which are related to abiotic stress. Besides, an *in silico* search for the previously described hormone-responsive *cis*-regulatory elements has been performed among the promoters of the family members from the high-affinity Cu transporters, termed COPT, in *Arabidopsis thaliana* and *Oryza sativa*. Finally, a model of the effect of putative modulators on target expression has been developed as a first step for deciphering the spatiotemporal codes for metalloprotein regulation in plants.

## High-Affinity Copper Influx in Plants

**FIGURE 1:**
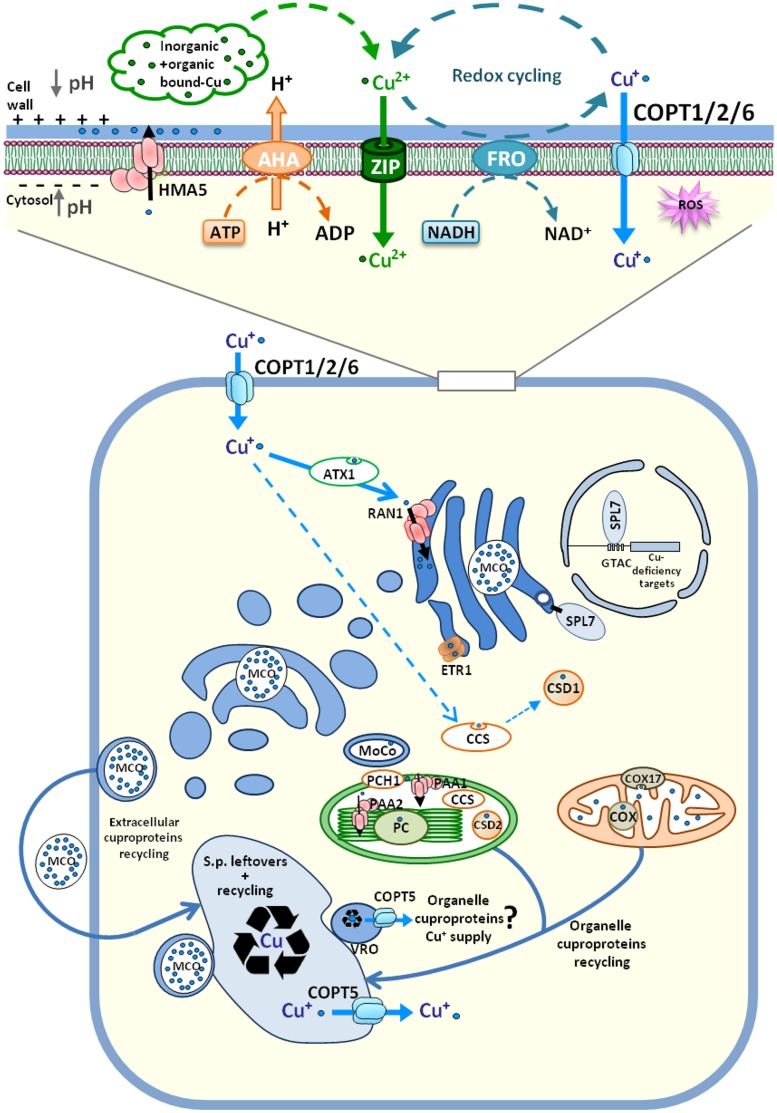
**Overview of *Arabidopsis thaliana* cellular Cu homeostasis**. Cu^+^ uptake through plasma membrane transporters COPT1/COPT2/COPT6 depends on the activity of AHA H^+^-ATPase and FRO cuproreductases. COPT-mediated Cu^+^ transport is coupled to metallochaperones transfer and its delivery to targets. Cuprochaperone CCS provides Cu^+^ to cytosolic superoxide dismutase CSD1. ATX1 transfers Cu^+^ to P-type ATPase RAN1, located at the ER, where Cu^+^ is probably acquired by cuproproteins, such as multicopper oxidases (MCOs), the ethylene receptor (ETR1), and the molybdenum cofactor (MoCo). The Cu resulting from recycling and from the secretory pathway leftovers converges into the vacuole or into vacuolar-related organelles (VROs). The Cu^+^ supply to chloroplasts and mitochondria can take place from the lumen through the COPT5 eﬄux function. See the main text for details. The direction of Cu^+^ traffic is indicated by arrows and Cu content is indicated by different intensities of blue.

Under aerobic conditions, Cu^2+^ is the most abundant form of copper in soil solution and probably enters plant root cells through divalent cation low-affinity transporters, such as some members of the ZIP family (ZIP2 and ZIP4; Wintz et al., 2003). However, this still has to be proven *in vivo*. Under metal deficiencies, plants acidify the external medium by using H^+^-ATPases (AHA; Santi and Schmidt, 2009). When Cu is scarce, plants use a Cu^+^-specific transport system based on Cu^2+^ reduction by plasma membrane NADPH-dependent cupric reductases FRO4 and FRO5 (Bernal et al., 2012) and on cytosolic uptake by high-affinity CTR-like transporters, denoted COPTs in plants (Sancenón et al., 2003; **Figure 1**). COPT substrate availability depends on both free external Cu (not bound to inorganic and organic complexes) and the Cu^+^/Cu^2+^ ratio according to external redox status conditions and the enzymatic activity of cuprooxidoreductases. The energetically expensive reductive strategy used for Cu^+^ uptake has been shown to be the predominant and ubiquitous mechanism for Cu acquisition in dicotyledons (Jouvin et al., 2012; Ryan et al., 2013). This redox strategy in Cu^+^ uptake could be an adaptation possibly required for specific high-affinity monovalent cation selection or/and for meeting specific Cu^+^ intracellular needs.

CTR-like Cu^+^ permeases function as trimers of small polypeptides. They have three transmembrane domains that contain the different conserved motifs involved in Cu^+^ binding from the extracytosolic compartment, translocation through the pore they form across the membrane and modulation of Cu^+^ delivery to metallochaperones for targeted distribution (Puig and Thiele, 2002; Pope et al., 2012). The aim of the present work is not to review currently available data on *Arabidopsis* COPT family members since they have been recently revised elsewhere (Peñarrubia et al., 2010; Puig, 2014). Briefly, *Arabidopsis* has a six-member family of *COPTs* (*COPT1-COPT6*). Each family member shows a tissue-specific expression pattern and performs specialized functions, as denoted by the phenotypes associated with *copt* mutant plants. Certain COPT proteins are regulated by Cu deficiency and are located in the plasma membrane (COPT1, COPT2, and COPT6), where they mediate Cu uptake from the external medium. COPT1 is expressed mostly in the root apex and pollen, where it participates in Cu^+^ uptake from soil and in Cu redistribution to reproductive organs (Sancenón et al., 2004). *COPT2* is the most expressed member of the family, is present in the root elongation zone and responds to both Cu and iron (Fe) deficiencies. *copt2* mutants are more resistant to double deficiency in Cu and Fe than wild type. The involvement of COPT2 in Fe metabolism could not result from a presumptive role in transporting Cu for a putative Cu-containing ferroxidase, which constitutes a common Fe–Cu connection in other organisms (Puig, 2014). Instead, a new and uncharacterized Cu–Fe crosstalk process has been suggested, where phosphate metabolism is also involved (Perea-García et al., 2013). COPT6 has been localized mainly at the vasculature of green tissues and reproductive organs where it can facilitate Cu redistribution under Cu scarcity (Jung et al., 2012; Garcia-Molina et al., 2013). Another COPT-type protein (COPT5) has been localized in the membrane of the prevacuolar/vacuolar compartment, where it is involved in the mobilization of Cu^+^ from the lumen to the cytosol in response to extreme Cu deficiency conditions (Garcia-Molina et al., 2011; Klaumann et al., 2011). COPT4 lacks the key methionine residues that are essential for Cu^+^ transport, which questions a possible role in Cu homeostasis. COPT3 is expressed at low levels and its function remains unsolved.

The characterization of the COPT family in rice (*O. sativa*), composed of seven members (*OsCOPT1–OsCOPT7*), uncovers the co-expression requirement of at least two family members to fully complement the yeast *ctr1Δctr3Δ* defect in high-affinity Cu uptake, except for *OsCOPT7* (Yuan et al., 2011). OsCOPT2, OsCOPT3, or OsCOPT4 have to cooperate with OsCOPT6 to mediate an efficient Cu transport, which is consistent with their physical interactions analyzed by the split-ubiquitin system (Yuan et al., 2011). On the other hand, with proteins OsCOPT1 and OsCOPT5, the cooperation with a third component, the MtN3/saliva-type protein XA13, is required to mediate the low-affinity Cu transport both in yeast and rice (Yuan et al., 2010). OsCOPT6 and OsCOPT7 relate more to *Arabidopsis* tonoplast located COPT5. However, the regulation of the different rice members by Cu deficiency and their tissue expression patterns do not allow predicting their intracellular locations. Bimolecular fluorescence complementation (BiFC) demonstrated that the *Vitis vinifera* AtCOPT5 homolog VvCTr1 monomers self-interact (Martins et al., 2014). Since according to the Irving–Williams series Cu has the highest capacity for binding to organic compounds, the endogenous concentrations of other metals, such as Fe, manganese (Mn), or zinc (Zn), may also influence Cu homeostasis by affecting *OsCOPTs* expression (Yuan et al., 2011), probably aimed at avoiding Cu competence under other metals scarcity. In this sense, during Zn limitation *Chlamydomonas reinhardtii* sequesters Cu in lysosome-related compartments and this strategy has been suggested to prevent Zn protein mismetallation by Cu when Zn is scarce (Hong-Hermesdorf et al., 2014).

Despite the prevailing dogma of protein–protein interactions mediating Cu^+^ delivery from transporters to target cuproproteins, under specific situations, such as adaptation to variable metal environmental levels or during diurnal fluctuations in transporters expression (see the *Temporal aspects in hormone and metal homeostasis* section), the OH^⋅^ damage derived from Cu^+^ traffic could affect the molecules adjacent to Cu^+^ transporters. Thus, COPT-mediated Cu^+^ traffic has been shown to produce rapid increases in Ca^2+^ influx and K^+^-eﬄux (Rodrigo-Moreno et al., 2013). Membranes and/or directly Ca^2+^/K^+^ channels could be OH^⋅^ targets, and could rapidly respond to Cu^+^ entrance and initiate subsequent signaling pathways where Ca^2+^, ROS and ABA play crucial roles (Gilroy et al., 2014). As a result, plants incorporate Cu^+^ under Cu deficiency by paying both a high energy cost and the subsequent damage caused by high Cu^+^ reactivity (Ravet and Pilon, 2013). Cu deficiency also causes increased oxidative stress in plants through photosynthetic electron transport chain (PETC) blockage at the essential cuproprotein plastocyanin level (Ravet and Pilon, 2013; Yruela, 2013). Although the specific signaling pathways of the diverse ROS produced at different cellular locations are starting to be identified (Gilroy et al., 2014), a complex scenario is envisaged under Cu deficiency since plant cells would experience the ROS signaling crosstalk deriving from both the metal scarcity at chloroplasts and that caused by an increased COPT-mediated Cu^+^-entrance to the plasma membrane.

## Intracellular Copper Traffic

Under non-stressed conditions, COPT-mediated Cu^+^ uptake is tightly coupled to its subsequent metabolic use. During this process, metallochaperones play an important role in transferring the metal between molecules, a process that kinetically competes with Cu^+^ dissociation in the solvent, which results in its immediate toxicity (Robinson and Winge, 2010; **Figure 1**). ATX1 is the cuprochaperone that delivers Cu^+^ to P-type ATPases (Cu^+^-ATPases), such as RAN1 located at the endoplasmic reticulum (ER). Subsequently, RAN1 pumps Cu^+^ into the lumen, where it is acquired by ER cuproproteins (Hirayama et al., 1999). Most extracellular and endomembrane cuproproteins follow the endocytic pathway to their final destinations and probably incorporate Cu upon transiting through the ER lumen or the trans-Golgi network. Thus, based on the relative abundance of cuproproteins, endocytic compartments are expected to display higher Cu levels than the nucleo-cytoplasmic space and, consequently, the main Cu^+^ flux should follow the COPT-ATX1-RAN1 pathway. In line with this, Cu delivery to cytosolic superoxide dismutase (Cu/ZnSOD) by the CCS cuprochaperone could represent a quantitatively minor Cu route (Robinson and Winge, 2010). Another Cu^+^-ATPase is HMA5. It is located at the plasma membrane, where it loads Cu^+^ into the xylem in roots and other organs which, under Cu excess conditions, functions in metal detoxification in both *Arabidopsis* and rice (Andrés-Colás et al., 2006; Kobayashi et al., 2008; Deng et al., 2013; **Figure 1**). Taken together, these facts predictably lead to Cu^+^ distribution between the nucleo-cytoplasmic and endocytic compartments by one or the other being favored, depending on the relative influx–eﬄux transport activity of COPT and Cu^+^-ATPases, respectively.

Chloroplasts are major consumers of Cu in plants, where it is incorporated into plastocyanin and Cu/ZnSOD, among other proteins (Ravet and Pilon, 2013). Once inside the chloroplast intermembrane space, the Cu^+^-loaded PCH1 cuprochaperone delivers Cu^+^ to the internal membrane-located P-type ATPase PAA1 (Blaby-Haas et al., 2014), which pumps Cu^+^ into the stroma (Shikanai and Fujii, 2013). PCH1 evolves by an alternative splicing event of the pre-mRNA encoding PAA1 (Blaby-Haas et al., 2014). Once inside the stroma, the CCS chaperone delivers Cu to chloroplastic Cu/ZnSOD and also to PAA2, which is the Cu^+^ P-type ATPase that delivers Cu to the thylakoids for plastocyanin supply (Abdel-Ghany et al., 2005; Blaby-Haas et al., 2014). The chloroplast caseinolytic protease (Clp) system is involved in specific PAA2 turnover under Cu excess in the stroma (Tapken et al., 2015). In mitochondria, Cu is required mainly for the assembly and activity of cytochrome c oxidase (COX) of the respiratory chain (Garcia et al., 2014). Cu delivery and insertion into COX is a complex process mediated by different metallochaperones present in the mitochondrial intermembrane space, such as COX17 (Balandin and Castresana, 2002; Attallah et al., 2007; **Figure 1**).

How Cu reaches organelles from an endosymbiotic origin, such as mitochondria and chloroplasts, is a poorly understood process. Since free Cu^+^ levels are extremely low in the cytosol (Rae et al., 1999), at least under Cu-limiting conditions, there must be other intracellular sources of Cu to ensure the arrival of Cu to organelles. Thus under nutrient deprivation, a putative Cu source could be the vacuole or vacuolar-related organelles (VROs). In these compartments, the valued metal arising from recycling metalloproteins and the scarce leftovers from secretory/endocytic pathways would converge (**Figure 1**). In addition, these compartments have been recently shown to participate in dynamic intracellular metal homeostasis (Blaby-Haas and Merchant, 2014; Hong-Hermesdorf et al., 2014). An increase in interorganellar communications with the membrane contact sites between mitochondria and the vacuole under nutrient deprivation stress has been recently described in yeast, which mainly serves for lipid and ion exchanges (Elbaz-Alon et al., 2014). If this were also the case in plants, organelles would be at “*the end of the line*” of the secretory pathway to acquire Cu which, under Cu deficiency, could lead to a competitive balance between previous Cu incorporation by in transit cuproproteins on the secretory pathway and the Cu leftovers available for the Cu supply of organelles.

## Regulation of Gene Expression under Copper Deficiency

Cu deficiency in plants induces the reprogramming of a number of metabolic processes, which represent an adaptive mechanism that has developed to survive under adverse conditions. The transcriptional response to Cu deficiency in *Arabidopsis* is mediated by a Zn finger transcription factor family member named SQUAMOSA-PROMOTER BINDING-LIKE PROTEIN 7 (SPL7). SPL7 is essential for the response to Cu deficiency *in vivo* through its binding to GTAC motifs in the promoters of target genes (Yamasaki et al., 2009; Bernal et al., 2012). The repression mechanism in the presence of Cu could be mediated by the displacement of Zn^2+^ by Cu^2+^ from the Zn fingers of the SPL7 transcription factor (Sommer et al., 2010). SPL7 has been recently shown to interact with KIN17, a conserved curved DNA-binding domain protein that promotes Cu-deficiency responses and alleviates oxidative stress responses, perhaps by preserving cell integrity and plant growth under Cu scarcity (Garcia-Molina et al., 2014a). SPL7 displays an operative transmembrane domain that has been shown to insert the protein into endomembranes, most probably at the ER. ER stress, as a result of Cu deficiency, also activates the SPL7 function by changing its location to the nucleus through its functional bipartite nuclear localization sequence (NLS) overlapping zinc-finger2 (ZF2) within the SPL7 SBP-domain. SPL7 dimerization adds another regulatory feedback mechanism to the ER-stress and SPL7-mediated Cu homeostasis response since it could affect its nuclear localization (Garcia-Molina et al., 2014b). These processes render SPL7 a crucial Cu sensor molecule in two topological spaces where Cu is initially distributed (the nucleo-cytoplasmic and the lumen of secretory pathway compartments). Thus, SPL7 could be able to perceive both ER stress, mediated by Cu deficiency through its C-terminal part, and Cu status in the nucleo-cytoplasmic space, through its SBP domain, maybe driving a converging and regulated response under Cu scarcity. This response could be even more complex if other components of the 16 SPL family members participate in the heterodimerization regulatory mechanism with SPL7 (Garcia-Molina et al., 2014b).

It is worth mentioning that the SPL7-mediated miRNA expression serves Cu redistribution in order to establish a priority ranking for Cu delivery to essential cuproproteins by avoiding Cu incorporation in non-essential proteins. Hence, one of the SPL7-mediated strategies used when Cu is limiting consists in replacing non-essential cuproproteins by other metalloproteins, usually Fe proteins, which play a similar role, probably in order to save Cu for essential cuproproteins, such as plastocyanin. With superoxide dismutases (SODs), substituting the Cu form (Cu/ZnSOD) with the Fe counterpart (FeSOD) is done by SPL7 under Cu-limited conditions by expressing FeSOD mRNA (*FSD1*) and miR398, since miR398 targets Cu/ZnSOD mRNAs (*CSD1* and *CSD2*) for degradation (Abdel-Ghany et al., 2005; Ravet and Pilon, 2013). Apart from metalloprotein substitution, another strategy for establishing priority ranking in Cu delivery under deficiency is the miRNA-mediated elimination of the first located cuproproteins on the pathway of Cu incorporation. In this case, other denoted Cu-miRNAs, such as miR408, target laccases (*LAC3, LAC12*, and *LAC13*; Yamasaki et al., 2007; Abdel-Ghany and Pilon, 2008). These cuproproteins predictably acquire Cu in the secretory pathway when transiting to their final destinations. Strikingly, miR408 accumulation and the subsequent down-regulation of the miR408 target genes in transgenic plants rescue developmental defects of the *spl7* mutant. This indicates that diminished Cu delivery to the endomembrane system to *en route* laccases redounds in increased Cu acquisition by plastocyanin at chloroplasts (Zhang and Li, 2013; Zhang et al., 2014). These results further reinforce the suggested hypothesis for chloroplasts’ Cu supply dependency on secretory pathway leftovers under Cu deficiency conditions. Accordingly, the miR408 strategy could consist in shortening “*the Cu metallation line*” by eliminating cuproproteins, located at the beginning of the spatiotemporal Cu delivery pathway, in order to allow further Cu delivery to other cuproproteins. Thus in this way, the scarce Cu atoms would arrive at the essential cuproproteins in the organelles situated later in the intracellular Cu delivery pathway. Besides acting at the intracellular level, miRNA-mediated Cu redistribution also affects the extracellular Cu content through changes in laccase expression and it could even act at the systemic level as a phloem-mobile long-distance signals in response to nutrient deprivation (Buhtz et al., 2010).

## Role of Copper Homeostasis in Hormone Biosynthesis and Perception

The role of Cu in hormone biosynthesis and perception has long since been known, mainly for its structural role in ethylene receptors (Rodríguez et al., 1999), and for molybdenum cofactor (MoCo) formation (Kuper et al., 2004) which is required for the biosynthesis of ABA and auxin indol-3-acetic (IAA) *via* indol-3-aldehyde, and because it is involved in the degradation of polyamines (PAs). More recent evidence has shown that Cu homeostasis also plays a prominent role on the salicylic acid (SA) signaling pathway (Wu et al., 2012; Yan and Dong, 2014) and is involved in ABA signal transduction as well as in the induction of nitric oxide (NO; Wimalasekera et al., 2011), an important and almost universal signaling molecule in plants.

As for other hormone-biosynthetic genes, data on the effect of Cu on ethylene production are accumulating. Thus, Cu excess has been reported to induce ethylene biosynthesis in broccoli seedlings (Jakubowicz et al., 2010) and in different organs of *Arabidopsis* plants (Arteca and Arteca, 2007), though this effect was not observed in the oldest leaves (Arteca and Arteca, 2007) or in seedlings (Lequeux et al., 2010). The Cu-inducible expression of ACC synthase (*ACS*) genes has been described in several species, such as potatoes, garden geraniums, and different tobacco cultivars (Schlagnhaufer et al., 1997). A range of Cu concentrations have also produced high ethylene levels, accompanied by toxicity symptoms in leaves and adventitious root formation in white poplars (Franchin et al., 2007). Therefore, in spite of some contrasting results, which may depend on the species or the organ studied, as well as on other factor such as the dosage and timing of the metal applied, it can be concluded that ethylene evolution is observed generally in response to Cu and other metals within a wide range of plant species (Maksymiec, 2007).

Five types of enzymes containing molybdenum (Mo) have been identified in plants to date. Among the plant enzymes containing MoCo, aldehyde oxidases merit special mention because several isoenzymes present a wide range of specificity for different aldehydes, and are also involved in the metabolism and signaling of different plant regulators. Thus *Arabidopsis* ALDEHYDE OXIDASE3 (AAO3) catalyzes the last step of ABA biosynthesis in conjunction with MoCo sulfurase (ABA3) and ALDEHYDE OXIDASE1 (AAO1) is involved in the biosynthetic pathway of auxins (Schwarz and Mendel, 2006). In these proteins, metal is bonded to pterins to form MoCo, whose biosynthesis has been widely studied and is closely related to the homeostasis of other metallic elements, such as Cu (Tejada-Jiménez et al., 2013). Although the role of Cu has not been fully elucidated, it is required for the activity of Cnx1G, an enzyme that catalyzes the insertion of Mo into molybdopterin. To date, this step of exchanging Cu and Mo appears to depend on unidentified cytoplasmic chaperones (Mendel and Kruse, 2012). In agreement with the Cu requirement for MoCo biosynthesis, increased Mo uptake has been observed under Cu deficiency in *Brassica napus* (Billard et al., 2014), although Cu treatment has been reported to inhibit *in vitro* MoCo biosynthesis (Kuper et al., 2004). This adds a second aspect to the interrelationship of Mo and Cu metabolisms, with Cu acting both positively and negatively during this process. In contrast, Cu-containing amine oxidases (CuAO) catalyze the oxidative de-amination of PA, such as putrescine and cadaverine, hence, degrading these plant regulators and affecting a number of the physiological processes they are involved in Cona et al. (2006).

In relation to the role of Cu in hormone perception and signaling, different components on both the ethylene biosynthesis and signal transduction pathways have been identified, some of which are differentially regulated depending on metal homeostasis, mainly Fe and Cu (Iqbal et al., 2013). The family of ethylene receptors (one of its members is ETR1) are cuproproteins where the Cu cofactor is necessary for hormone perception (Rodríguez et al., 1999). Further evidence for the importance of this metal on the ethylene-signaling pathway emerged with the discovery that *EIN2*, a central signal transducer on the ethylene-signaling pathway, has a significant homology to NRAMP divalent cation transporters (Alonso et al., 1999). It has been shown that both ETR1 and EIN2 are also involved in some ABA responses, and serve as integration nodes between both hormones signaling pathways (Wilkinson and Davies, 2010). In this context, it is also interesting to note that CuAO1 contributes to ABA- and PA-induced NO biosynthesis, and also influences ABA signal transduction (Wimalasekera et al., 2011).

In turn, SA is involved mainly in the systemic-acquired response (SAR), which is considered a plant immune response to pathogens. Transcriptional coregulator NONEXPRESSOR OF PATHOGENESIS-RELATED GENES1 (NPR1) activates the expression of SA-dependent defense genes, and Cu ions have been reported to be involved in its binding to SA in plants (Wu et al., 2012). NPR1 is a redox-regulated signaling protein and SA activates thioredoxin, which leads to NPR1 reduction, thus converting it into active monomers that are translocated from the cytosol to the nucleus by activating the defense gene expression (Tada et al., 2008). NPR1 and its homologs NPR3 and NPR4 are SA receptors (Pajerowska-Mukhtar et al., 2013). NPR1 functions in the crosstalk between SA and jasmonic acid (JA) signaling and is modulated by ethylene (Leon-Reyes et al., 2009), which indicates that NPR1 is a key player in integrating redox, metal and hormonal signaling (see the *Putative modulators of the hormonal and copper homeostasis crosstalk* section).

As previously indicated, although Cu is required for hormone biosynthesis and perception, an excess of this metal is not always associated with an increased accumulation or a higher sensitivity to the hormone but with toxicity symptoms. Indeed, under Cu excess, complex signal transduction networks produced by different hormonal interactions are involved in the morphological responses induced by Cu and other heavy metals. Auxins, ethylene, and ROS have been identified as major components of these morphological alterations (Potters et al., 2009) and in *Arabidopsis*, NO has been also shown to participate in the Cu excess-induced responses (Xiong et al., 2010; Peto et al., 2011). However, some results contradict one another probably due to differences in the concentration of the heavy metal applied, the treatment conditions, the age of the plant and the variety of tissues examined. In this sense, our current understanding of the molecular mechanisms involved in heavy metal toxicity and their interactions with phytohormones is quite limited and requires further studies.

## Effects of Phytohormones on the Copper High Affinity COPT Transporters

Little is known about how hormones influence Cu homeostasis to adapt nutrient availability to the growth requirements while avoiding its toxic effects. Due to the scarce literature at this respect, as an approach to address hormone influence on Cu homeostasis we have undertaken an *in silico* search of the putative hormone-responsive elements in the promoters of the high-affinity COPT transporters in both *A. thaliana* and *O. sativa* (**Table 1**). The most abundant hormone-responsive elements present in the promoters from *COPT* family members were those involved in ABA and gibberellin (GA) signaling. While in *Arabidopsis* the highest number corresponded to GA-related elements, the opposite occurred in *Oryza*, in which the number of ABA-related elements was highest. Ethylene and auxin presented a lower number of *cis*-elements, similar in both species. JA-related elements were the less abundant in both *Arabidopsis* and *Oryza*, although this number was larger in rice (**Table 1**).

**Table 1:** Analysis of the putative hormone-responsive *cis*-elements in the COPTs promoters.

		*Arabidopsis thaliana*						*Oryza sativa*							
Hormones	Motifs	*COPT1*	*COPT2*	*COPT3*	*COPT5*	*COPT6*		*COPT1*	*COPT2*	*COPT3*	*COPT4*	*COPT5*	*COPT6*	*COPT7*	
ABA	ABRE	4	3	–	3	8	18	8	5	9	4	3	8	3	40
	DPBF	1	–	1	2	2	6	5	–	–	2	2	1	1	11
	DREB	–	–	–	1	–	1	–	–	–	–	–	–	1	1
	MYB-MYC BOX	6	6	2	1	4	19	3	1	5	6	1	1	1	18
	Others	–	–	–	–	1	1	–	–	3	–	2	3	1	9
	**Total**	**11**	**9**	**3**	**7**	**15**	**45**	**16**	**6**	**17**	**12**	**8**	**13**	**7**	**79**
GA	CARE	–	1	–	1	1	3	2	1	–	–	–	–	–	3
	GARE	1	5	8	2	2	18	–	1	–	–	2	–	–	3
	WRKY	3	4	9	9	9	34	6	7	13	7	3	7	5	38
	Others	3	1	3	3	2	12	1	1	2	–	3	–	–	7
	**Total**	**7**	**11**	**20**	**15**	**14**	**67**	**9**	**10**	**15**	**7**	**8**	**7**	**5**	**51**
Ethylene	ERE	–	1	–	–	–	1	–	–	–	–	–	–	1	1
	WBOX ERF3	2	4	4	4	7	21	2	4	5	1	1	3	2	18
	Others	1	–	–	1	–	2	–	1	1	–	1	2	–	5
	**Total**	**3**	**5**	**4**	**5**	**7**	**24**	**2**	**5**	**6**	**1**	**2**	**5**	**3**	**24**
AUXIN	ASF1	–	3	3	3	1	10	2	1	2	1	1	2	–	9
	SAUR	–	–	–	–	–	–	1	–	1	1	–	–	1	4
	SURE	–	–	1	6	1	8	2	–	1	1	1	1	3	9
	Others	–	–	–	4	–	4	–	1	–	–	–	–	–	1
	**Total**	**–**	**3**	**4**	**13**	**1**	**22**	**5**	**2**	**4**	**3**	**2**	**3**	**4**	**23**
JA	GCC CORE	–	–	–	–	–	–	1	1	–	–	7	3	–	12
	T/G BOX	1	–	–	–	–	1	1	–	–	–	–	–	–	1
	**Total**	**1**	–	–	–	–	**1**	**2**	**1**	–	–	**7**	**3**	–	**13**

*The COPTs genomic sequences from *Arabidopsis thaliana* and *Oryza sativa* were obtained from the phytozome database. The promoter sequences were defined as the 1,000 bp upstream of the five prime untranslated region (5^′^-UTR) from the corresponding COPTs genes. The *in silico* analysis of the *cis*-elements present in the promoter regions was performed in the PLACE database. The commonest and most abundant *cis*-elements are shown for each hormone, including ABRE (ACGTGG/TC), DPBF (ACACNNG), DREB (A/GCCGACNT), MYB (CNGTTR), MYC (CANNTG), CARE (CAACTC), GARE (TAACAAA), WRKY (C/T)TGAC(T/C), ERE (AWTTCAAA), WBOX ERF3 (TGACY), ASF1 (TGACG), the SAUR motif (CATATG), SURE (GAGAC), the GCC core (GCCGCC) and T/G BOX (AACGTG). All the other *cis*-elements are included in the “Others” group.*

The *cis*-acting elements and *trans*-acting factors involved in ABA-induced gene expression have been extensively analyzed and the main ABA responsive *cis*-elements are ABRE (ABA-responsive element), MYB/MYC and DPBF (Dc3-Promoter Binding Factor; Nakashima et al., 2014). The ABRE motifs have been identified in the promoter region of ABA-inducible genes and several basic leucine zipper (bZIP) proteins have been shown to bind these motifs (Fujita et al., 2011). The presence of ABRE motifs in the *Arabidopsis COPTs* promoters of the members located at the plasma membrane (**Table 1**) could be related to an early response to dehydration in vegetative parts with dark-induced senescence, as suggested by Simpson et al. (2003). The presence of drought- and ABA-related MYB and MYC motifs, mostly in the *COPTs* promoter members of the *Arabidopsis* plasma membrane-located transporters (**Table 1**), also suggests that these transporters could be regulated under developmental or stressful conditions by increasing ABA content. DPBF and DREB (dehydration responsive element binding) motifs, which have been described to play an important role in the ABA response in seeds and during early seedling establishment stages (Lopez-Molina and Chua, 2000), though scarce, are present in most *Arabidopsis* *COPTs* members (**Table 1**).

In the dehydration response context, it is worth noting that ABA plays an important role in the regulation of the stomatal behavior and gas exchange of dehydrated plants and, hence, in long-distance transport of nutrients. The effects of ABA on H^+^-ATPases in guard cells are well-known during stomata closure, and may involve Ca^2+^, light and ROS signaling (Brault et al., 2004; Zhang et al., 2004a). Ca^2+^ is located both up- and downstream of ABA signaling in guard cells, and Cu induces a peak in Ca^2+^ uptake and an increase in ROS (Rodrigo-Moreno et al., 2013). The convergence of both signaling pathways suggests that the ABA-mediated drought response and long-distance transport of nutrients might influence Cu homeostasis to some extent. Romero et al. (2012) reported that an ABA-deficient orange mutant, which is prone to fruit dehydration, was unable to induce early molecular responses to moderate water stress observed in wild-type fruit. Among the transcriptomic responses described, *Citrus* *COPT1*, *COPT2*, and *COPT5* transporters were induced in parental, but not in the ABA-deficient fruit. Other di- and trivalent inorganic cation transporters were also induced only in parental fruit under those conditions, such as the *Citrus* Fe transporters *IRT1*, *NRAMP1*, *NRAMP3*, and *FERRITIN*. These results support the idea of an ABA/drought-mediated regulation of the genes involved in these metals homeostasis. Further research would be necessary to unravel whether the response to stresses increasing endogenous ABA levels in *Arabidopsis* plants also involves the regulation of these metal transporters. Within this context, studies including ABA-deficient plants, such as the *aba2* mutant, would be even more explanatory. Nevertheless, the presence of ABA-related elements in the promoter sequences of *COPT*s in both *Arabidopsis* and *Oryza* (**Table 1**) points to a common regulation of these genes among different species.

Gibberellin is an essential phytohormone that controls many aspects of plant development and its antagonist role with ABA is well-known (Weiss and Ori, 2007). The WRKY family is one of the largest transcription factor families involved in GA and ABA responses, and in defense against pathogens and senescence in *Arabidopsis* (Eulgem et al., 2000). Accordingly, many putative *cis*-elements are present in *COPTs* promoters in both *Arabidopsis* and *Oryza* (**Table 1**). Most published WRKY proteins bind to the cognate *cis*-acting element containing the W-box in the promoter (Xie et al., 2005). For instance, *OsWRKY71* is highly expressed in rice aleurone cells, where it represses the GA-induced *Amy32bα*-amylase promoter (Zhang et al., 2004b). The GA response GARE *cis*-element and the pyrimidine box (included in “*Others”*; **Table 1**) are partially involved in sugar repression in rice embryos (Morita et al., 1998). These GARE elements are required in *Arabidopsis* to modulate endogenous GA concentrations during seed germination (Ogawa et al., 2003).

ETHYLENE INSENSITIVE3 (EIN3) is a transcription factor that binds to the promoters of the target genes denoted *ETHYLENE RESPONSIVE FACTORS* (*ERFs*). ERFs might also play a role in ABA signaling, and they encode transcription factors that bind to the promoters containing a GCC motif by either activating or repressing target genes (Nakano et al., 2006). Ethylene WBOX ERF3 is the prevalent *cis*-element in *COPTs* promoters (**Table 1**). ERFs are often involved in the transcription of the defense genes that encode antifungal proteins, including chitinases and glucanases, in response to ethylene and fungal elicitors (Nishiuchi et al., 2004). Since differential Cu sensitivity between plants and fungal pathogens is often used for Cu-based fungicide treatments, the characterization of the interaction between ethylene signaling and Cu homeostasis could be relevant for optimizing fungal infected crops defense responses.

Among other environmental stimuli, root growth and development is highly dependent on nutrient availability. Changes in the root architecture have been reported at different nutrient levels, mainly phosphate, sulfate and nitrate, with specific effects on primary and lateral roots that are dependent on each nutrient (López-Bucio et al., 2003; Tian et al., 2014). However, responses to micronutrient levels, such as Cu, have also been observed (Kazan, 2013). Available data indicate that auxins seem to be the main hormonal mediators of root responses at soil nutrient levels. According to Lequeux et al. (2010), Cu excess remodelates root auxin distribution, and thus affects the mitotic activity of the meristem. Yuan et al. (2013) reported that this Cu-induced auxin redistribution involves the eﬄux carrier PIN1, which is responsible for root acropetal auxin transport. Spatio-temporal asymmetric auxin distribution has been indicated as a means of coordinating plant development (Tanaka et al., 2006). Auxin *cis*-elements have a similar distribution in both *Arabidopsis* and *Oryza*. SURE and ASF1 are the prevalent elements (**Table 1**) and are related to sulfur transport in the root epidermis and cortex cells (Maruyama-Nakashita et al., 2005). The SAUR motif is frequent in *Oryza*, but not in *Arabidopsis* (**Table 1**). This *cis*-element may play a significant role in secondary growth, especially secondary phloem and bark formation (Oh et al., 2003).

Cu is an essential nutrient for reproductive development, mainly due to diminished pollen viability under Cu scarcity, considered a typical symptom of Cu deficiency (Marschner and Marschner, 2012), although other effects, such as anther dehiscence and filament elongation, contribute to male fertility decrease. Accordingly, *COPTs* have been shown to be highly expressed in pollen and the anther filament (Sancenón et al., 2004; Garcia-Molina et al., 2011; Jung et al., 2012; Gayomba et al., 2013; Perea-García et al., 2013), and lack of COPT1 produces pollen defects (Sancenón et al., 2004). JA appears to be a critical signal for anther dehiscence, but other hormones, such as auxins and gibberellins, are also involved (Wilson et al., 2011). The JA receptor degrades a jasmonate-ZIM domain protein repressor (JAZ) of the JA responsive genes (Wasternack and Hause, 2013). This repressor binds to specific MYC and MYB transcription factors (Wager and Browse, 2012). Thus MYB21 and MYB24 function as direct targets of JAZs to regulate male fertility in *A. thaliana* (Song et al., 2011). Despite JA motifs being scarce in *COPTs* promoters, a GCC core element is present in four genes of the *Oryza* transporters, although not in *Arabidopsis.* This element, originally described in a gene encoding a plant defensin, is commonly used as a marker for the characterization of JA-dependent defense responses (Brown et al., 2003). JA, together with SA and ethylene, constitutes the main defense line against biotic stresses in plants. JA has been shown to act as a potent inducer of the expression of CuAO, induced by wounding and antagonizing with SA and ABA. Their action is mediated by oxidative stress since the wounding induction of CuAO leads to H_2_O_2_ production (Rea et al., 2002). Interestingly, the Cu-sensitive rice pathogen *Xanthomonas oryzae pv oryzae* (*Xoo*) strain exploits *O. sativa* Cu^+^- transport mechanisms through COPT1 and COPT5 to remove Cu from xylem vessels, where *Xoo* multiplies and spreads to cause disease (Yuan et al., 2010). So the different Cu sensitivity exhibited by distinct organisms suggests that modification of Cu homeostasis can be used by plants to fight pathogens.

## Temporal Aspects in Hormone and Metal Homeostasis

Plant growth and development are regulated by the clock, hormonal changes and nutrient signals, suggesting a complex reciprocal relationship between the clock and metabolic signaling processes (Bolouri Moghaddam and Van den Ende, 2013). Understanding temporary regulation is essential to highlight the dynamic aspects in the homeostasis of transition metals and their integration with other processes. The central oscillator temporarily orders biological processes to adapt them to daily environmental cycles of light and temperature through a wide variety of regulatory mechanisms (Seo and Mas, 2014). Ninety percent of *Arabidopsis* genes exhibit diurnal or circadian regulation, and at least 30% of the transcriptome is regulated by the circadian clock (Michael et al., 2008). Most hormones fluctuate during light/dark cycles, which leads to a significant enrichment of circadian-regulated hormone-responsive genes (Nováková et al., 2005). Microarray analyses indicate that the transcripts involved in hormonal metabolism, perception and signaling are regulated by the circadian clock, which gates hormonal signals to better adapt daily physiological changes (Robertson et al., 2009). In turn, different phytohormones influence distinct plant circadian clock parameters, a fact that evidences a reciprocal interaction between hormone signaling and the clock. Whereas brassinosteroid shortens clock periodicity, ABA prolongs the circadian period in a light-dependent manner. Cytokinins delay the circadian phase and auxins regulate circadian amplitude and clock precision. Accordingly, hormone mutants exhibited predictable clock phenotypes (Hanano et al., 2006).

Ethylene biosynthesis is clock-regulated and peaks at the subjective day, which correlates with increased *ACC SYNTHASE 8* (*ACS8*) transcript levels, subjected to circadian clock control. However, ethylene mutants do not alter circadian rhythms (Thain et al., 2004). Certain *ACS* and *ACC OXIDASE* (*ACO*) genes, which participate in ethylene biosynthesis, are circadianly regulated with a similar phase to ethylene emissions. Ethylene signaling components, such as *EIN3*, also display a similar oscillatory pattern of expression in response to light and sugar availability (Yanagisawa et al., 2003; Lee et al., 2006). *XAP5 CIRCADIAN TIMEKEEPER* (*XCT*) is also involved in blue light-dependent ethylene responses in *Arabidopsis* shoots (Ellison et al., 2011).

The expression of ABA- and JA-responsive genes also oscillates diurnally (Mizuno and Yamashino, 2008). The genes implicated in the synthesis of geranylgeranyl diphosphate (GGDP), an intermediate metabolite in isoprenoids synthesis, which leads to the production of chlorophylls, carotenoids, tocopherols, ABA and GA, are clock-regulated. Five of these genes peak in the subjective morning, as do ABA metabolic genes *NINE-CIS-EPOXYCAROTENOID DIOXYGENASE3* (*NCED3*) and *ABA DEFICIENT2* (*ABA2*). ABA levels fluctuate with diurnal rhythms in different species, and a significant overlap has been reported between the genes induced by either ABA or JA and the genes that oscillate with light/dark cycles. More than 40% of ABA-induced genes are circadianly regulated, and most peak in the subjective morning, in accordance with ABA levels (Covington et al., 2008). A number of ABA signaling genes also fluctuate during the day cycle. Thus several ABA receptors, some PP2CA-type constitutive repressors, the positive effectors of the ABA signaling pathway, such as SnRK2s and some members of the canonical ABF transcription factors, display diurnal cycles (Seung et al., 2012). It is noteworthy that unlike the above-described ABA biosynthetic genes, those involved in ABA signaling rarely peak at the same time, not even those that belong to the same protein family, and they display overlapping functions (Seung et al., 2012). The circadian clock regulates the diurnal expression of ABA-related gene *ABAR/CHLH/GUN5* through TOC1 directly binding to its promoter (Legnaioli et al., 2009). TOC1 is a key repressor of gene expression (Huang et al., 2012). Conversely, ABA treatment induces *TOC1* at midday, the subsequent down-regulation of clock gene expression and the lengthening of the free running clock period reveal a feedback loop that reciprocally links *ABAR* and *TOC1* expressions (Legnaioli et al., 2009; Pokhilko et al., 2013). ABI3 also physically interacts with TOC1 (Kurup et al., 2000; Dekkers et al., 2008). Taken together, these results suggest that the ABA response is gated by the circadian clock for the fine tuning of environmental fluctuations in water availability, which occur mainly at midday (Seung et al., 2012).

The temporal aspects of Cu homeostasis are starting to be elucidated (Andrés-Colás et al., 2010; Peñarrubia et al., 2010; Perea-García et al., 2010). The requirements of metals vary during the diurnal cycles of light and darkness since photosynthesis is the process with the greatest metals requirements. To reconcile the dynamic changes between supply and demand, there are complex homeostasis networks whose aim is to maintain adequate metal levels in different tissues and developmental stages (Puig et al., 2007; Palmer and Guerinot, 2009; Puig and Peñarrubia, 2009). The fact that plasma membrane *COPTs* are regulated by Cu status through SPL7 has led to hypothesize that an intracellular oscillation of Cu is feasible since, under Cu-deficient conditions, induced plasma membrane COPT members are involved in metal uptake until sufficient Cu levels are reached and their expressions are inhibited. This self-regulatory feedback loop of Cu on its own transporters expression can cause oscillation in COPT expression driving to a cycling Cu concentration (Peñarrubia et al., 2010). Accordingly, *Arabidopsis COPT1* and *COPT2* transcripts are circadianly regulated (Haydon et al., 2011). If, as recently suggested, SPL7-mediated Cu deficiency stress responses can be perceived in the ER lumen (Garcia-Molina et al., 2014b), oscillations in Cu levels can take place in this compartment and perhaps at other subcellular locations, which depend on the endomembrane system for Cu supply. In this sense, cycling Cu uptake fluxes might drive the complex Cu muﬄing processes that take place in the entire cell.

Experimental data reinforce the interconnection between Cu homeostasis and the circadian rhythms in organisms other than plants (Perea-García et al., 2010). Recently, Yamada and Prosser (2014) reported the effects of Cu availability on the circadian clock of suprachiasmatic nuclei (SCN) in humans, where it appears to modulate the phase through glutamate signaling. In several animal systems, hormones, such as pineal-secreted melatonin, are connected with the circadian clock (Gamble et al., 2014). Melatonin has metal-chelating properties, which may contribute to reduce metal-induced toxicity (Romero et al., 2014). The wide distribution of melatonin in biological systems, including plants (Arnao and Hernández-Ruiz, 2014), delineates a putative hormonal, metal and clock interconnection that deserves further characterization. On the other hand, although the physiological and molecular mechanisms are obviously different in each group of organisms, the interconnections of the circadian clock with metals and hormones seem to be conserved. Plants are excellent models to study the effects of metal homeostasis on the circadian rhythms due to the widely presence of clocks in the different cells and tissues of vegetal organisms and the variety of well-established circadianly regulated processes.

Like hormones, different metals distinctly affect circadian clock parameters. The oscillation amplitude of the gene expression of two of the main components of the central oscillator, *CIRCADIAN CLOCK ASSOCIATED1* (*CCA1*) and *LATE ELONGATED HYPOCOTYL* (*LHY*), increases under Cu deficiency, but their period remains mostly unaffected (Andrés-Colás et al., 2010). Magnesium (Mg) shortage also affects *CCA1* and *LHY1* expression, which increases at the end of the light period (Hermans et al., 2010). In contrast, Fe deficiency provokes period lengthening and Fe transport forms an interconnected loop with the central oscillator (Chen et al., 2013; Hong et al., 2013; Salomé et al., 2013; Tissot et al., 2014). It has been suggested that phytochromes and the functional state of chloroplasts would participate in a new retrograde Fe sensor-dependent route, which constitutes a central oscillator loop (Salomé et al., 2013).

Due to the pervasive oscillating nature in the expression of ion channels and nutrient transporters, the circadian signal spreads and, in turn, the nutritional status affects the circadian clock (Ko et al., 2009; Haydon et al., 2011). This mutual interaction between nutrient homeostasis and circadian clock components integrates temporary information and rhythmically coordinates, and also optimizes metabolism and physiology (Sanchez et al., 2011; Haydon et al., 2013). Therefore, understanding the mechanisms that continuously adjust the circadian clock is essential for improving plant fitness (Hotta et al., 2007). If the mutual influence between metal homeostasis and circadian rhythms is a widespread fact among higher eukaryotes, a wide range of new experimental approaches in plants is foreseen to address important biological questions, where plant research could facilitate the analysis of the temporal dimension contribution to metal-dependent cell processes.

## Putative Modulators of the Hormonal and Copper Homeostasis Crosstalk

**FIGURE 2:**
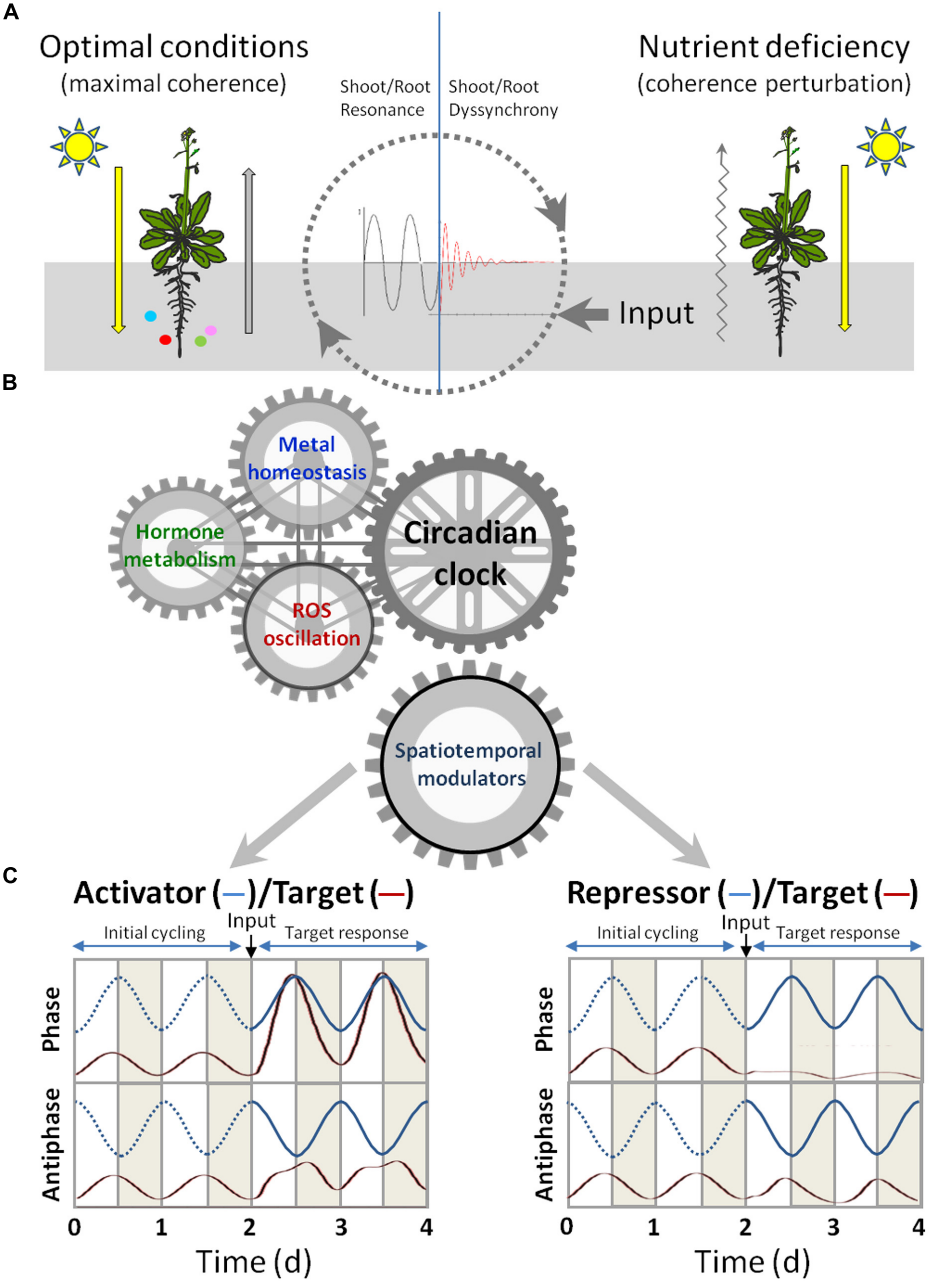
**Signaling crosstalk among circadian clock, hormones and metal homeostasis. (A)** Under optimal conditions (left), light signaling influences the nutritional responses in roots and signals from adequate nutrient supply are coordinated with light signaling to drive maximal coherence between roots and shoots development. Under nutrient deficiency (right), a coherence perturbation is produced. **(B)** The circadian clock is a key integrator of environmental processes, such as light cycles and nutrient availability, and of endogenous cycles, such as ROS oscillation and hormone metabolism. The aim of the circadian clock-mediated regulation of spatio-temporal modulators is to optimize plant growth under both optimal cycles and altered conditions. **(C)** Model for the effect of activators and repressors on target gene expression. The time course of *mRNA* accumulation from modulators’ (blue lines) target genes (red lines) under circadian control is shown throughout four daily cycles (12 h light in white and 12 h dark in gray). The circadian oscillating levels of the activator (left) and repressor (right), running in phase (upper panel) or in antiphase (lower panel) with their targets, are shown for two cycles. The dotted lines indicate lack of modulator activity. The effects on the target expression of an input (indicated by an arrow) that activates the modulator is shown during the next two cycles. The changes underwent by the mRNA levels of the target genes are modeled by the differential equation shown in the main text. The numerical integration of Eq. 1 (with auxiliary Eq. 2) was performed by the COPASI program (Hoops et al., 2006) with σ = 0 (*q* = 0) for time 0–2 (i.e., the first two cycles) and with σ = 0.125 (*q* = 1.5) from time 2 onward. The values for other constants were *A* = 12, α = 5.

In this section we seek the putative spatiotemporal regulators that are the best candidates for the integration of signaling from circadian and/or light cycles, ROS, hormones and Cu homeostasis. These regulators, either activators or repressors, can respectively enhance or attenuate the gene expression of multiple target genes at different levels. Here we emphasize mainly the transcriptional and post-transcriptional regulation that contributes to temporarily adapt their functions (**Figure 2**).

In previous sections, some putative modulators, such as NPR1 or melatonin, and their integrative functions in light, metal, hormonal and redox processes have been described. Other relevant candidates for these integrative functions are considered herein. Among them we find zinc finger (ZF) proteins, both classical transcriptional regulators, including Cu deficiency master regulator SPL7 and non-classical ZF, that act at the post-transcriptional level (Lee and Michel, 2014). In these metallo-regulatory proteins, metals ions can serve as “switches” during their nucleic acid recognition process. In fact, ZF proteins are potential targets for toxic metals, which may affect their structure and function (Hartwig, 2001). By way of example, it has been suggested that excess Cu^2+^ disrupts the glycine-rich proteins containing the RNA recognition motifs that are essential for post-transcriptional regulation and may impair the development of plants or animals (Qin et al., 2014). Although ZF proteins are usually identified also by the presence of similar sequence elements, most provide no *in vivo* evidence for the native metals ions that they bind and about the physiological consequences of metal substitution. In certain ZF proteins, transition metal coordination has been reported to be associated with rapid oxidation through radical formation by Fenton chemistry (Lee and Michel, 2014). This fact, in addition to the inability of metal binding by oxidized Cys residues, makes these proteins putative candidates for integrating metal and oxidative stress responses. ROS produced by metal deficiencies or during metal trafficking could act as potent intermediates in the crosstalk between metal homeostasis and the circadian clock. Indeed photosynthetic organisms cyclically suffer from the oxidative stress derived from light utilization in chloroplasts, and ROS cycling is integrated into the circadian clock. In fact peroxiredoxin oxidation–reduction cycles constitute a universal marker for circadian rhythms in all domains of life, including *Arabidopsis* (Edgar et al., 2012), and the genes involved in the synthesis of those compounds that prevent ROS production are clock-regulated and peak near subjective dawn (Lai et al., 2012). Consequently, it is predictable that, whenever possible, other oxidative processes could be temporarily separated from the intense light period in order to avoid both the saturation of antioxidative plant system capacity and interference with other ROS signaling pathways.

Recent research is uncovering the key integrators between light and hormone pathways, which may be also influenced by metal status. Among the factors that link the light signaling to GA and ABA antagonistic signaling we find PHYTOCHROME INTERACTING FACTOR3-LIKE5 (PIL5), a basic helix-loop-helix protein. The interaction of PIL5 with phytochromes induces its degradation through the 26S proteasome and promotes seed germination (Oh et al., 2006). PIL5 up-regulates GA and down-regulates ABA levels by modulating the expression of their metabolic genes (Seo et al., 2009). This regulation is mediated by non-classical ZF protein TZP (Tandem Zinc Protein) SOMNUS (Kim et al., 2008) which could be affected by Cu levels providing a putative link between metal homeostasis and hormone signaling. Another TZP protein, termed Cth2, is considered a master regulator of the stability of many target mRNAs to mediate global metabolic remodeling in *Saccharomyces cerevisiae* when Fe is scarce (Sanvisens et al., 2011). Cth2 is conserved in yeast, human and plants, which points to TZP proteins as putative mediators between hormones and metal homeostasis.

bHLH transcription factor ELONGATED HYPOCOTYL 5 (HY5) has been shown to integrate the light, clock and hormones, mainly auxin and cytokinin, signaling pathways by promoting the expression of negative regulators and by controlling the protein stability of HY5, respectively (Cluis et al., 2004; Vandenbussche et al., 2007). HY5 physically interacts with core clock component CCA1 (Andronis et al., 2008). In fact HY5 binds to ∼40% of the coding loci in the *Arabidopsis* genome, including miR408, a Cu-deficiency target involved in the post-transcriptional degradation of its target multicopper oxidases Laccase-type (*LAC12* and *LAC13*; Lee et al., 2007; Zhang et al., 2011). It is noteworthy that SPL7 and HY5 have been shown to physically interact and co-regulate the expression of a large cohort of genes, including miR408, which integrate both transcriptional and post-transcriptional regulations in response to light and Cu status (Zhang et al., 2014). In addition, the sulfate assimilation regulatory circuit and glutathione accumulation are transcriptionally regulated by light and cross-interact with ABA (Cao et al., 2014). A possible interplay of HY5 with another transcription factor has been shown to participate in the regulation of sulfate assimilation by light (Koprivova et al., 2014). Interactions between light and hormone signaling pathways have long been observed, being HY5 one of the main integrators (Lau and Deng, 2010). HY5 binds the G-box (CACGTG) and G-box-related *cis*-elements as well as other factors, some of them related with ABA signaling (Wind et al., 2013). A competence in binding to G-box-related elements could drive a crosstalk between light and hormonal signaling. In this sense, HY5 interaction with the Cu deficiency master regulator SPL7 would participate in this competence, where the arrangement of Cu-deficiency and G-box-like *cis*-elements in the promoters of target genes could play an important role. Taken together, these results indicate that HY5 is a central, temporal integrator that adapts plant development to cycling nutrient homeostasis, hormone signaling and light/dark cycles, and that it is also a spatio-temporal modulator of multiple targets for oscillatory adaptation to changing environmental cues.

MicroRNAs are also key candidates for dynamic and long-distance abiotic stress signaling and ABA induce the accumulation of a number of small RNAs, including miR168. Its exclusive target is ARGONAUTE1 (AGO1), a key component of the RNA-inducing silencing complex. AGO1 alterations have been involved in ABA sensitivity, although the exact mechanism remains unclear (Earley et al., 2010). Nonetheless, much is known about how AGO1 is regulated by the miR168-mediated feedback loop during ABA responses. ABRE-binding transcription factors ABF (1–4) activate *miR168a* expression through the ABRE *cis*-element present in its promoter (Li et al., 2012). As mentioned before, several Cu-miRNAs are responsive to Cu-deficiency and govern the priority ranking for Cu-acquisition that ABA-dependent AGO1 regulation can alter. Since miR398 controls antioxidant activities, in addition to Cu-deficiency, other processes involving enhanced ROS regulate its expression (Zhu et al., 2011). Dynamic miR398 expression regulation under drought stress remains a controversial matter as it hinders assigning a clear role for ABA during the process (Ding et al., 2013).

Another key issue is how metal status signals, wherever perceived, are intracellularly communicated with the nucleus. A putative candidate for this communication is AP2-type transcription factor ABI4, which plays a central role in mediating mitochondrial and chloroplast retrograde signaling (Koussevitzky et al., 2007; Giraud et al., 2009; Wind et al., 2013). ABI4 was originally characterized for its role in ABA signaling (Finkelstein et al., 1998). ABA biosynthesis and signaling extensively interact with sugars. ABI4 directly regulates plastidial Cu^+^-ATPase PAA1 through binding to the *PAA1* promoter. In fact, *PAA1* and *ABI4* expressions are mutually affected and lack of the PAA1 function provokes insensitivity to high glucose. It has been suggested that PAA1 functions in the bidirectional communication between the plastid and nucleus due to its essential plastid activity, and perhaps affects ROS, sugar levels, or even both (Lee et al., 2012).

During the evolution of eukaryotic organisms, including higher plants, the ability to modulate cycling changes in intracellular compartmentalized metal concentrations and metal traffic and eﬄux, denoted as metal muﬄing, had to be coordinated with other cyclic responses to environmental and endogenous cues for a certain developmental coherence to be achieved. Light is the main environmental signal for plant development, which is perceived in shoots, but also influences root growth. Nutrient uptake takes place in roots, but is regulated by light conditions. During these long-distance signaling processes, the crosstalk among ROS, hormones and the circadian clock are known to play a crucial function whose aim is to coordinate a temporary coherent developmental program between roots and shoots. Under optimal environmental conditions, global plant growth presents maximal coherence, whereas non-optimal conditions drive to different degrees of stress, depending on the severity and length of stress. Root abiotic stress, such as metal deficiency, can be considered to distort maximal coherence (**Figure 2A**).

The interplay among ROS, hormones and the circadian clock, and metal influence, during all these processes could play a key role in temporal responses to stress. In order to recover coherence, the effect of spatio-temporal modulators, acting at different levels of gene expression on their respective targets, should be considered to understand the dynamics of attaining plant fitness under stress conditions (**Figure 2B**). Modulators can be divided into activators and repressors by increasing or decreasing the expression of their target genes, respectively. Frequently, the expression of both modulators and their target genes are subjected to circadian regulation. Hence, their expression will be oscillatory with the same period (i.e., 1 day) but with a certain phase difference. For simplicity we will consider only two situations (phase and antiphase) to illustrate these interactions (**Figure 2C**). The following differential equation was used to model the change in the amount of target mRNA (m) in time (t, in days) when a specific signal or input, such as deficient metal supply, triggered the expression of the modulator:

(1) dm/dt=A⋅[1+sin⁡(2⋅π⋅t)]⋅M−α⋅m⁢

where A is a positive constant that represents the circadianly regulated transcriptional strength, and α is the first-order kinetic constant for mRNA degradation. The effect of the modulator was introduced by the factor M. If the modulator is an activator driven by the circadian clock, then M equals B defined as:

(2) B=1+q⋅[1+sin⁡(2⋅π⋅t+f)]

where f is the phase-shift with the target (e.g., f = 0 for phase, f = π for antiphase) and q represents the strength of the modulator effect on the transcriptional rate of the target. Thereby, the oscillatory expression of the activator will act as a multiplicative factor on the target mRNA synthesis term in Eq. 1. On the other hand, if the modulator is a repressor, then M equals 1/B and the oscillatory expression of the repressor will attenuate the synthesis of the target mRNA. The relative strength of either modulator may be expressed by σ = q/A, which is the ratio of the modulator to circadian transcriptional strengths (see the legend of **Figure 2C** for further details of the parameter values). If the modulator is an activator, the phase synchrony between the activator and its target genes will redound on “*constructive interference*,” in analogy to the physical concept of waves adding up their amplitudes when in phase. As a result, the transcription of a target gene that oscillates with the same phase as the activator will be enhanced (**Figure 2C**). On the other hand, the target genes that oscillate in anti-phase with activators will display only a moderately increased and somewhat distorted transcriptional response. In contrast, the temporal phase synchrony between a repressor and its target gene will result in “*destructive interference*” (i.e., waves annihilating each other when interacting with opposite phases), which will lead to gross attenuation, or even the suppression, of the target’s oscillatory behavior, while the genes that shift to anti-phase will be much less affected by the repressor (**Figure 2C**). This oversimplified model emphasizes the importance of the initial relative phase of modulators and their target genes in the response to an input that triggers the modulator function. This mathematical approach can also help understand the differential target responses based on interactions under the cyclic gene expression of both modulators and their targets. Moreover, this model predicts that changes in the modulators phase would profoundly impact on their target responses and consequently these wave interferences can serve to modulate and integrate endogenous responses in order to improve plant fitness under stress conditions.

In summary, these observations highlight the importance of understanding the synchronization of the endogenous circadian system with environmental, nutritional and hormonal factors. Disrupted orchestration of circadian, nutritional, and hormonal rhythms occurs readily through environmental and/or genetic perturbations, and results in a state of circadian dyssynchrony. Chronic dyssynchrony ensues if endogenous hormonal or/and nutritional cycles are frequently altered, and modulators do not have the time required to complete resynchronization with environmental cycles. Impairment of the normal circadian rhythmicity produced by aberrant metabolic homeostasis could negatively affect a plethora of biological processes and drive to a misalignment of metabolic cycles with the central oscillator. If modulators of the circadian clock mechanism and their role in the integration with hormonal and nutrient cycles are identified, the prospect of targeting these mechanisms in plants offers much potential.

## Conflict of Interest Statement

The authors declare that the research was conducted in the absence of any commercial or financial relationships that could be construed as a potential conflict of interest.
